# Investigation on CMB monopole and dipole using blackbody radiation inversion

**DOI:** 10.1038/s41598-023-30414-4

**Published:** 2023-02-27

**Authors:** Somita Dhal, R. K. Paul

**Affiliations:** Department of Physics, Birla Institute of Technology, Mesra, Ranchi, Jharkhand 835215 India

**Keywords:** Cosmology, Cosmology

## Abstract

The COBE/FIRAS dataset is used to calculate the Cosmic Microwave Background temperature and the uncertainty using the Blackbody Radiation Inversion (BRI) method. In this research work, the procedure is somewhat comparable to the mixing of weighted blackbodies in the case of the dipole. The temperature and its spreading for the monopole and dipole, respectively, are 2.741 ± 0.018 K and 2.748 ± 0.270 K. This dipole spreading exceeds the spreading predicted by taking relative motion into account (i.e., 3.3 × 10^−3^ K). The comparison of the probability distributions for the monopole spectrum, dipole spectrum, and their resultant is also displayed. It is shown that the distribution is symmetrically orientated. We estimated the µ and y-distortions by interpreting the spreading as the distortion and found that they are of the order of 10^−4^ and 10^−5^, respectively, for the monopole spectrum and 10^−2^ for the dipole spectrum. The paper also highlights the effectiveness of the BRI method and hints at future applications in the thermal nature of the early universe.

## Introduction

The existence of a universal, thermal radiation field had been predicted clearly by 1948 and was laid forgotten until 1965^1^. A signal which was initially detected in 1965 was reportedly being emanated from every direction of the sky and came to be known as CMB^2^. Two major contributions made by COBE/FIRAS are:—the detection of the CMB thermal spectrum and its anisotropies^3^. The COBE/FIRAS detected the first thermal spectrum and observed that the obtained experimental results are similar to the spectrum of a blackbody at T = 2.728 K. After the detection, much research has been conducted on cosmic microwave background radiation^4–10^. The radiation is first predicted to be isotropic and homogeneous but later studies proved it to be anisotropic^11–14^. The study of CMB has put forth some noteworthy propositions on the genesis of the extensive and expansive formations in the universe^15^. According to the most recent research and observations on the CMB, the radiation observed was caused by the mixing of black bodies with different temperatures rather than a single blackbody with a particular temperature. This causes the µ and y—distortion in the CMB spectrum^16,17^. Several theories have been put forward for the calculation of µ and y—distortion^18–21^. The most important parameters for determining the genesis of the cosmos are temperature and CMB distortions. We can analyse the probability distribution of temperature for the CMB since the CMB spectrum resembles a Planck spectrum. One such technique is blackbody radiation inversion which has been proposed to solve the probability distribution of temperature^22–25^. A short history of dipole measurement up to the time of the COBE satellite is reported in the paper^26^. After that much research has been done on CMB dipole^27–30^. Recently a new blackbody inversion method is being employed to study the monopole portion of the CMB and the temperature derived is 2.69 K with an uncertainty of 0.195 K^31^. The measured spectral distortions in that method for monopole are 10^−2^ for µ-type and 10^−3^ for y type. The values are very imprecise than values |µ|< 9 × 10^−5^ and |µ|< 1.5 × 10^−5^ as per prior report^6^. In the present article, we used the technique of Blackbody Radiation Inversion (BRI) with a choice of new distribution function to calculate the temperature, spreading, µ and y—distortions from both monopole and dipole spectrum. The main advantage of the present BRI technique over existing methods is its simplicity. It requires only 3 data points to produce a probability distribution of temperature. This distribution shows how blackbodies of different temperatures are present in the spectrum, and their contribution is measured using the weight factor. This introduces a process of mixing blackbodies, and we observe them as y and µ-distortions.

The obtained temperature for monopole and dipole with uncertainty is 2.741 ± 0.018 K & 2.748 ± 0.270 K, respectively. In this process, we found some weighting factors multiplied by the monopole formula to get the dipole. It is shown that the process is somewhat comparable to the mixing of weighted blackbodies. Both monopole and dipole spectrum intensities are reconstructed to validate the accuracy of this choice of the probability function. The |y| and |µ| distortions are calculated and the obtained values are of order 10^−5^ and 10^−4^ respectively for the monopole spectrum. The |y| and |µ| distortions values for the dipole spectrum are in the order 10^−2^. Furthermore, this method can be used in infrared remote sensing calibration as the precise measurement temperature is crucia1^32,33^.

The article is categorized into four parts. Sections "Method" 2(a) and 2(b) describe the methodology for obtaining the cosmic microwave background temperature and uncertainties for both monopole and dipole spectrum respectively. Also, it describes the method of obtaining the reconstructed intensities along with y and µ distortions for both spectrums. In Section "Discussion" we discuss the implication of the result. Section "Summary" summarizes the result.

## Method

### (a) For CMB monopole

A blackbody is defined as an object perfectly capable of absorbing all incident radiation on it of any frequency. Planck's radiation law relates the intensity of radiation produced at a given temperature to either frequency or wavelength^34,35^.

1 $$\text{B}\left( \nu \right) = \frac{2 \text{h} \nu ^{3} }{\text{c}^{2} }\frac{1}{\text{e}^{\frac{\text{h}\nu }{\text{kT}}} - 1}$$

where h, T, k, c, ν are Planck’s constant, absolute temperature, Boltzmann’s constant, speed of light (in vacuum) and frequency respectively. The total radiated power for the monopole spectrum can be seen as the integration over the spectral radiance w.r.t. temperature as in Eq. (2).

2 $${\text{W}}_{{\text{m}}} \left( \nu \right) = \frac{{2{\text{h}}\nu ^{3} }}{{{\text{c}}^{2} }}\int\limits_{0}^{\infty } {\frac{{\alpha \left( {\text{T}} \right)}}{{\left( {{\text{e}}^{{\frac{{{\text{h}}\nu }}{{{\text{kT}}}}}} - 1} \right)}}} {\text{dT}}$$

The semi-infinite integral considers all possible values of temperature. But here we have assumed that the temperature of the blackbody varies in an interval of T_1_ to T_2_ as the spectrum of CMB has a resemblance with a blackbody spectrum of temperature 2.728 K.

3 $${\text{W}}_{{\text{m}}} \left( \nu \right) = \frac{{2{\text{h}}\nu ^{3} }}{{{\text{c}}^{2} }}\int\limits_{{{\text{T}}_{1} }}^{{{\text{T}}_{2} }} {\frac{{\alpha \left( {\text{T}} \right)}}{{\left( {{\text{e}}^{{\frac{{{\text{h}}\nu }}{{{\text{kT}}}}}} - 1} \right)}}} {\text{dT}}$$

The section of sky observed by the telescope comprises different blackbody radiators in thermal equilibrium with each other at a temperature T. In Eq. (2), α(T) is introduced as the probability distribution of temperature and its dimension is $\frac{1}{{\text{K}}}$. The experimental values for W_m_(ν) are available^6^. Furthermore, using these data, the value of α(T) can be calculated. Based on previous experimental data^6^; the spectrum of CMB has a resemblance with a blackbody spectrum of temperature 2.728 K. The width of a gaussian distribution at half of its maximum value is 2.35σ. For a reasonable choice of σ = 0.8 and T = 2.73 K; T_2_ = 2.73 + $\frac{1}{2}$ × 0.8 × 2.35 = 3.67 K and T_1_ = 2.73 − $\frac{1}{2}$ × 0.8 × 2.35 = 1.79 K. So, it is reasonable to take the range between T_1_ = 1 K to T_2_ = 6 K for considering beyond 1σ.

4 $${\text{W}}_{{\text{m}}} \left( \nu \right) = \frac{{2{\text{h}}\nu ^{3} }}{{{\text{c}}^{2} }}\int\limits_{1}^{6} {\frac{{\alpha \left( {\text{T}} \right)}}{{\left( {{\text{e}}^{{\frac{{{\text{h}}\nu }}{{{\text{kT}}}}}} - 1} \right)}}} {\text{dT}}$$

For mathematical appropriacy and simplicity, ${\text{G}}_{{\text{m}}} \left( \nu \right) = \frac{{{\text{c}}^{2} }}{{2{\text{h}}\nu ^{3} }}{\text{W}}_{{\text{m}}} \left( \nu \right)$ is to be employed. G_m_(ν) is dimensionless. Hence,

5 $${\text{G}}_{{\text{m}}} \left( \nu \right) = \int\limits_{1}^{6} {\frac{{\alpha \left( {\text{T}} \right)}}{{\left( {{\text{e}}^{{\frac{{{\text{h}}\nu }}{{{\text{kT}}}}}} - 1} \right)}}} dT$$

To solve this integration in Eq. (5), employing the change of variable T = T_1_ + (T_2_–T_1_) t, Eq. (5) becomes^25^

6 $$  \mathop {{\text{G}}_{{\text{m}}} \left( \nu  \right){\mkern 1mu} = \left( {{\text{T}}_{2} - {\text{ T}}_{1} } \right)}\int \limits_{0}^{1} \frac{{\upalpha \left( {\left( {{\text{T}}_{2} - {\text{T}}_{1} } \right){\text{t}} + {\text{ T}}_{1} } \right)}}{{\left( {{\text{e}}^{{\frac{{{\text{h}}\nu }}{{{\text{k}}\left( {\left( {{\text{T}}_{2} - {\text{T}}_{1} } \right){\text{t}} + {\text{ T}}_{1} } \right)}}}} - 1} \right)}}{\text{dt}} $$

Recently a blackbody radiation inversion technique is proposed^31^ to solve this type of integral using an analytical function a(t) = $\sinh \left( {{\text{p}}^{2} {\text{t}}} \right).{\text{m}}.{\text{e}}^{{ - {\text{nt}}^{2} }}$. The measured spectral distortions in that method are 10^−2^ for µ-type and 10^−3^ for y-type. The values are very imprecise than values |µ|< 9 × 10^−5^ and |µ|< 1.5 × 10^−5^ as per prior report^6^. So, to meet the expectation with accuracy a new analytical gaussian function is proposed in this present article as z(t).

7 $${\text{z}}\left( {\text{t}} \right) = {\text{m}}.{\text{e}}^{{ - \left( {\frac{{\left( {{\text{t}} - {\text{n}}} \right)^{2} }}{{{\text{p}}^{2} }}} \right)}}$$

The nature of the probability distribution is expected to be close to Gaussian. So, a gaussian distribution is chosen in Eq. (7). So,

8 $$ {\text{G}}_{{\text{m}}} \left( \nu \right){\mkern 1mu} = \left( {{\text{T}}_{2} - {\text{ T}}_{1} } \right)\int\limits_{0}^{1} {\frac{{{\text{z}}\left( {\text{t}} \right)}}{{\left( {{\text{e}}^{{\frac{{{\text{h}}\nu }}{{{\text{k}}\left( {\left( {{\text{T}}_{2} - {\text{T}}_{1} } \right){\text{t}} + {\text{ T}}_{1} } \right)}}}} - 1} \right)}}} {\text{ dt}}$$

This well-motivated choice of taking z(t) as a gaussian probability density with its peak position (n), FWHM (p) and overall amplitude (m); is to calculate the temperature and distortions with accuracy which is described in the discussion section. Here the process of finding ${\upalpha }\left( {\left( {{\text{T}}_{2} - {\text{T}}_{1} } \right){\text{t}} + {\text{ T}}_{1} } \right)$ is equivalent to finding z(t). Here in Eq. (7) m, n and p are three determinable parameters. The experimental value of G_m_(ν) is given by,

9 $$  {\text{G}}_{{\text{m}}} \left( \nu  \right){\mkern 1mu}  = \frac{{2{\text{h}}\nu ^{3} }}{{{\text{c}}^{2} }} \times {\text{ I}}_{{\text{m}}}  $$

The experimental values of ${\text{I}}_{{\text{m}}}$ are given^6^. The change of variable T = T_1_ + (T_2_–T_1_) t is used to solve the integral in Eq. (5) and T_1_ = 1 K & T_2_ = 6 K is chosen. So, ${\text{t }} = \frac{{{\text{T}} - {\text{T}}_{1} }}{{{\text{T}}_{2} - {\text{T}}_{1} }} = \frac{{{\text{T}} - 1}}{5}$. We are intending to find the distribution ${\upalpha }\left( {\text{T}} \right)$; So, the probability function is written in terms of T. Equation (7) becomes,

10 $${\text{z}}\left( {\text{T}} \right) = {\text{m}}.{\text{e}}^{{ - \left( {\frac{{\left( {\left( {\frac{{{\text{T}} - 1}}{5}} \right) - n} \right)^{2} }}{{p^{2} }}} \right)}}$$

Now taking the Eq. (8) in L.H.S. and the calculated G_m_(ν) from Eq. (9) in the R.H.S, a set of three equations are obtained for a corresponding set of three frequencies, which are then mathematically simulated and corresponding values of m, n, p is obtained. Furthermore, this process is similarly repeated for a triple set of three more frequencies and the values are tabulated below.

For each set of frequencies, the corresponding m, n and p values are calculated and are put in Eq. (10) to obtain four probability distributions of temperature. For each frequency set the values of m, n, p and corresponding probability functions are listed in Table 1.

**Table 1:** Values for various probability functions are given, and they are identified by the notations as f(T), v(T), g(T) and n(T), which correspond to various sets of frequencies.

Frequency set (× 10^11^ Hz)	m	n	p	Probability functions
3.402, 3.54, 3.675	21.432593364347	0.346933782481	0.005186207337232	f(T)
2.586, 2.724, 2.859	22.407436083304	0.347869646661	0.004983003283835	v(T)
1.089, 1.224, 1.362	30.933771151701	0.348954457249	0.003589324373322	g(T)
1.497, 1.635, 1.77	15.32444593428	0.34887929187	0.007206065989433	n(T)

The average of the above probability distributions is denoted as X(T).

11 $${\text{X}}\left( {\text{T}} \right) = \frac{{{\text{f}}\left( {\text{T}} \right) + {\text{v}}\left( {\text{T}} \right) + {\text{g}}\left( {\text{T}} \right) + {\text{ n}}\left( {\text{T}} \right)}}{4}$$

**Figure 1:**
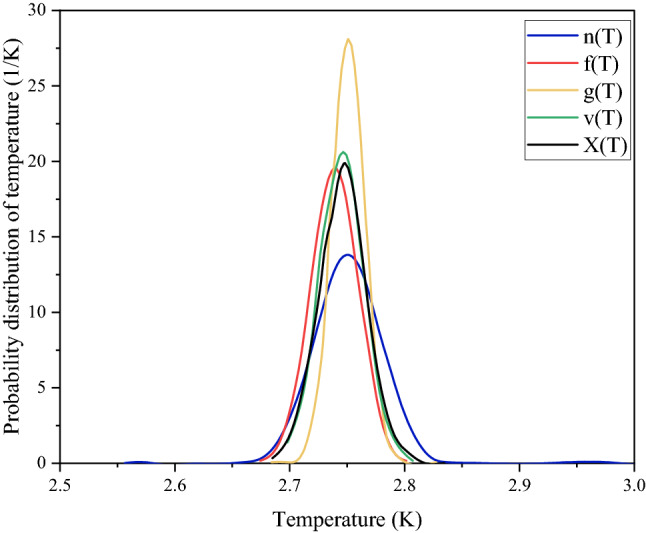
Four different probability functions f(T), v(T), g(T) and n(T) along with their resultant X(T) are plotted against absolute temperature.

The figure below shows the four different probability functions along with the average function. Although the integration range is from 1 to 6 K, for a clear view of the graph in Fig. 1 the x-scale is taken from 2.5 K to 3 K.

The average probability distribution is to be normalized in the temperature range of 1 K to 6 K. The normalization constant is -

12 $$\frac{1}{{\int_{1}^{6} {\text{X}} \left( {\text{T}} \right){\text{dT}}}} = 1.016$$

The final normalized probability distribution of temperature is denoted as α_m_(T).

13 $$\upalpha _{{\text{m}}} \left( {\text{T}} \right){\mkern 1mu} = 1.016 \times {\text{ X}}\left( {\text{T}} \right)$$

We calculate the ‘first order moment’ or the ‘mean normalized temperature’ for monopole and denoted as ${\text{T}}_{{{\text{mean}}}}^{{{\text{monopole}}}}$.

14 $${\text{T}}_{{{\text{mean}}}}^{{{\text{monopole}}}} = 1.016\int\limits_{1}^{6} {\text{T}} .{\text{ X}}\left( {\text{T}} \right){\text{dT }} \cong 2.740{\text{ K }}$$

The ‘second order moment’ or ‘spreading’ is calculated as

15 $$\sigma ^{2} = 1.016\int\limits_{1}^{6} {\left( {{\text{T}} - 2.74} \right)^{2} } {\text{ X}}\left( {\text{T}} \right){\text{ dT}} = 3.493 \times 10^{{ - 4}}$$

From this, the uncertainty in temperature σ =  ± 0.018 K.

**Figure 2:**
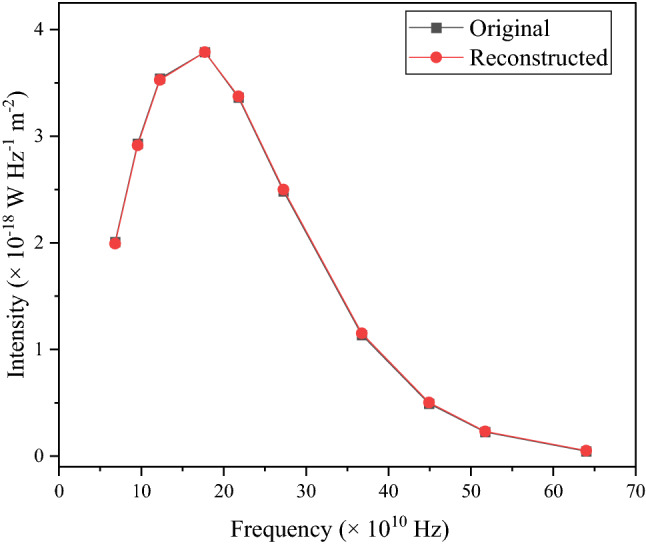
The original input intensity data for the monopole spectrum is reconstructed using the obtained probability distribution of temperature ${\upalpha }_{{\text{m}}}$(T). Plots of experimental and reconstructed intensities versus frequency are shown. The figure shows a close resemblance between the reconstructed data and the original data.

In order to test the precision of this method for determining the probability distribution of temperature for monopole spectrum; for various frequency ν, using the calculated ${\upalpha }_{{\text{m}}}$(T) in Eq. (4), we were able to reconstruct the radiation intensity. Figure 2 shows the comparison of COBE/FIRAS original spectrum data with reconstructed data.

The values of frequency, original and reconstructed intensities are listed in Table 2.

**Table 2:** All the values of original and reconstructed intensities for the monopole are listed.

Frequency (× 10^10^ Hz)	Original intensity (× 10^−18^ W Hz^−1^ m^−2^)	Reconstructed intensity (× 10^−18^ W Hz^−1^ m^−2^)
6.81	2.00723	1.993
9.54	2.93024	2.914
12.24	3.54081	3.527
17.7	3.78901	3.788
21.78	3.36278	3.373
27.24	2.48239	2.500
36.75	1.13568	1.152
44.91	0.49223	0.503
51.72	0.22644	0.2329
63.99	0.04523	0.0514

The chi-square is calculated using the following formula^36^.

16 $$\upchi ^{2} = \sum {{\text{w}}_{{\text{i}}} } \left[ {({\text{R}}_{{\text{i}}} - {\text{O}}_{{\text{i}}} } \right)]^{2}$$

where, ${\upchi }^{2} { }$ = Chi squared.

${\text{R}}_{{\text{i}}} =$ Reconstructed set of data points.

${\text{O}}_{{\text{i}}}$ = Original data points.

${\text{w}}_{{\text{i}}}$ = 1/${\upsigma }_{{\text{i}}}^{2}$ where ${\upsigma }_{{\text{i}}}$ denote the error on the data point.

The values of ${\upsigma }_{{\text{i}}}$ are available in the paper^6^. The reduced chi-square $\left( {\frac{{{\upchi }^{2} }}{NDF}} \right)$ is calculated to be 1.55. Now, to calculate the resulting temperature after mixing; this formula ${\text{T}}_{{{\text{new}}}}^{{{\text{monopole}}}} = {\text{ T}}\left[ {{1} + \left( {\frac{{{\Delta T}}}{{\text{T}}}} \right)^{2} } \right]$ is used^18^. ${\text{T}}_{{{\text{new}}}}^{{{\text{monopole}}}} = { 2}.{\text{741 K}}$ taking our result of ${\text{T}}_{{{\text{mean}}}} = {2}.{74}0{\text{K}}$ and ΔT = 18 mK. The formula used in the literature^18^ for calculating the y and µ distortions is used for calculating µ and y distortions. The µ and y distortions are computed here as $\mu = { 2}.{8 } \times \left( {\frac{{{\Delta T}}}{{\text{T}}}} \right)^{2} = {1}.{2}0{8} \times {1}0^{{ - {4}}}$ and ${\text{y }} = \frac{1}{2} \left( {\frac{{{\Delta T}}}{{\text{T}}}} \right)^{2} = { 2}.{157} \times {1}0^{{ - {5}}}$ The order of distortion in this article is consistent with the prior values of distortions reported as |µ|< 9 × 10^−5^ and |y|< 1.5 × 10^−5^.

### (b) CMB dipole

This method is also used to calculate the temperature and spreading for the dipole spectrum and to compare how the spreading is changing for the dipole with respect to the monopole. The total radiated power for the dipole spectrum can be seen as the derivative of spectral radiance w.r.t. temperature which is multiplied by the dipole amplitude as in the Eq. (17).

17 $${\text{W}}_{{\text{d}}} \left( \nu \right) \, = {\text{T}}_{{\text{amp }}} \frac{{{\text{dB}}}}{{{\text{dT}}}} = {\text{I}}_{{\text{d}}}$$

where, T_amp_ = 3.369 × 10^−3^K.

So, by differentiating B with respect to T and multiplying T_amp_ and introducing α(T) as the probability distribution of temperature and taking the integral from 1 to 6 K we get,

18 $${\text{W}}_{{\text{d}}} \left( \nu \right) = \frac{{2{\text{h}}\nu ^{3} }}{{{\text{c}}^{2} }}\int\limits_{1}^{6} {\frac{{{\text{e}}^{{\frac{{{\text{h}}\nu }}{{{\text{kT}}}}}} \frac{{{\text{h}}\nu }}{{{\text{kT}}^{2} }}\alpha \left( {\text{T}} \right)}}{{\left( {{\text{e}}^{{\frac{{{\text{h}}\nu }}{{{\text{kT}}}}}} - 1} \right)^{2} }}} {\text{T}}_{{{\text{amp}}}} {\text{dT}}$$

Breaking down Eq. (18) we get,

19 $${\text{W}}_{{\text{d}}} \left( \nu \right) = \frac{{2{\text{h}}\nu ^{3} }}{{{\text{c}}^{2} }}\int\limits_{1}^{6} {\frac{{{\text{e}}^{{\frac{{{\text{h}}\nu }}{{{\text{kT}}}}}} \frac{{{\text{h}}\nu }}{{{\text{kT}}^{2} }}}}{{\left( {{\text{e}}^{{\frac{{{\text{h}}\nu }}{{{\text{kT}}}}}} - 1} \right)}}} \frac{1}{{{\text{e}}^{{\frac{{{\text{h}}\nu }}{{{\text{kT}}}}}} - 1}}{\text{T}}_{{{\text{amp}}}} \upalpha \left( {\text{T}} \right){\text{ dT}}$$

From Eq. (1) and (19)

20 $$\mathop \int \limits_{1}^{6} {\text{B}}\left( \nu \right){\text{R}}\left( \nu \right) {\upalpha }\left( T \right){\text{dT }} = {\text{ I}}_{{\text{d}}}$$

We can say Eq. (20) gives the mixing of weighted Planckian or Blackbodies.

Here ${\text{R}}\left( \nu \right){\mkern 1mu} = \frac{{{\text{e}}^{{\frac{{{\text{h}}\nu }}{{{\text{kT}}}}}} \frac{{{\text{h}}\nu }}{{{\text{kT}}^{2} }}}}{{\left( {{\text{e}}^{{\frac{{{\text{h}}\nu }}{{{\text{kT}}}}}} - 1} \right)}}$ T_amp_ is the weight factor. It is a dimensionless parameter.

Here we utilised the process of blackbody radiation inversion for finding the probability distribution of temperature for superposition or mixing of weighted blackbodies. The CMB's blackbody radiation field is inverted using the inversion process in order to determine the distribution of temperature of the inducing medium. The procedure is similar to that used in monopole.

So, for dipole taking the chosen probability distribution z(t); Eq. (19) can be written as,

21 $${\text{G}}_{{\text{d}}} \left( \nu \right){\mkern 1mu} = \left( {{\text{T}}_{2} - {\text{ T}}_{1} } \right){\text{ T}}_{{{\text{amp}}}} \int\limits_{0}^{1} {\frac{{{\text{e}}^{{\frac{{{\text{h}}\nu }}{{{\text{k}}\left( {\left( {{\text{T}}_{2} - {\text{T}}_{1} } \right){\text{t}} + {\text{ T}}_{1} } \right)}}}} \frac{{{\text{h}}\nu }}{{{\text{k}}\left( {\left( {{\text{T}}_{2} - {\text{T}}_{1} } \right){\text{t}} + {\text{ T}}_{1} } \right)^{2} }}{\text{ z}}\left( {\text{t}} \right)}}{{\left( {{\text{e}}^{{\frac{{{\text{h}}\nu }}{{{\text{k}}\left( {\left( {{\text{T}}_{2} - {\text{T}}_{1} } \right){\text{t}} + {\text{ T}}_{1} } \right)}}}} - 1} \right)^{2} }}} {\text{ dt}}$$

and,

22 $${\text{G}}_{{\text{d}}} \left( \nu \right) = \frac{{2{\text{h}}\nu ^{3} }}{{{\text{c}}^{2} }} \times {\text{ I}}_{{\text{d}}}$$

The experimental values of I_d_ are given^6^. Now taking the Eq. (21) in L.H.S. and the calculated G_m_(ν) from Eq. (22) in the R.H.S, a set of three equations are obtained for a corresponding set of three frequencies, which are then mathematically simulated and corresponding values of m, n, p is obtained. Furthermore, this process is similarly repeated for a triple set of three more frequencies and the values are given in Table 3.

**Table 3:** Values for various probability functions are given, and they are identified by the notations as x(T), d(T), l(T) and q(T), which correspond to various sets of frequencies.

Frequency set (× 10^11^ Hz)	m	n	p	Probability functions
3.402, 3.54, 3.675	1.879960727754	0.351772488661	0.056329541255	x(T)
2.586, 2.724, 2.859	1.43111413211	0.349811525032	0.077487885366	d(T)
1.089, 2.586, 3.402	1.493990697668	0.329975168162	0.081457672699	l(T)
1.497, 1.635, 1.77	1.406770742674	0.347708368285	0.081597126116	q(T)

For each set of frequencies, the corresponding m, n and p values are calculated and are put in Eq. (10) to obtain four probability distributions of temperature.

The average of the above probability distributions is denoted as A(t).

23 $${\text{A}}\left( {\text{T}} \right){\mkern 1mu} = \frac{{{\text{x}}\left( {\text{T}} \right) + {\text{d}}\left( {\text{T}} \right) + {\text{l}}\left( {\text{T}} \right) + {\text{ q}}\left( {\text{T}} \right)}}{4}$$

**Figure 3:**
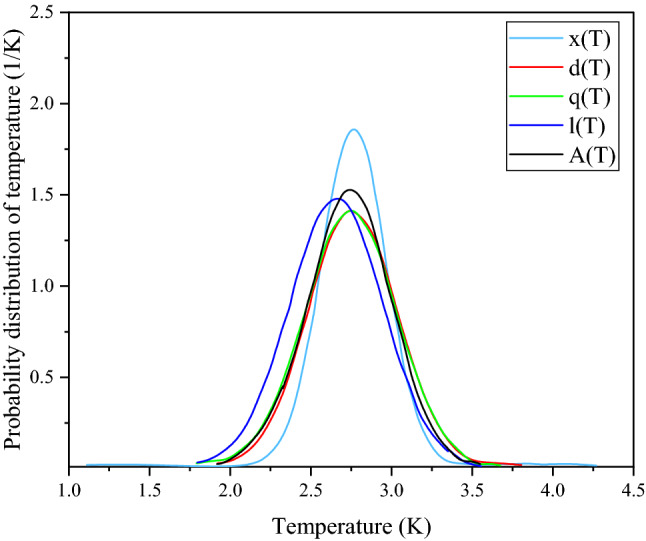
Four different probability functions x(T), d(T), q(T) and l(T) along with their resultant A(T) are plotted against absolute temperature. The scales are taken considering the clear view of the graph.

The four probability distributions i.e., x(T), d(T), l(T), q(T) for each frequency set along with their average probability A(T) is shown in Fig. 3.

The average probability distribution is to be normalized in the temperature range of 1 k to 6 k. The normalization constant is -

24 $$\frac{1}{{\int_{1}^{6} {\text{A}} \left( {\text{T}} \right){\text{dT}}}} = {\mkern 1mu} 0.996$$

The final normalized probability distribution of temperature is denoted as α_d_ (T).

25 $${\upalpha }_{{\text{d}}} \left( {\text{T}} \right) \, = \, 0.{996} \times {\text{ A}}\left( {\text{T}} \right)$$

We calculate the ‘first order moment’ or the ‘mean normalized temperature’ for the dipole and denoted it as ${\text{T}}_{{{\text{mean}}}}^{{{\text{dipole}}}}$.

26 $${\text{T}}_{{{\text{mean}}}}^{{{\text{dipole}}}} = 0.996\mathop \int \limits_{1}^{6} {\text{T}}.{\text{ A}}\left( {\text{T}} \right){\text{dT }} \cong { }2.722{\text{ K }}$$

The ‘second order moment’ or ‘spreading’ is calculated as,

27 $$\sigma^{{2}} = 0.996{ }\mathop \int \limits_{1}^{6} \left( {{\text{T}} - 2.722} \right)^{2} {\text{ A}}\left( {\text{T}} \right){\text{ dT}} = \, 0.0{73}$$

From this, the uncertainty in temperature for dipole σ =  ± 0. 270 K.

Here also to test the precision of this method for determining the probability distribution of temperature for the dipole spectrum we reconstructed the dipole spectrum with our calculated ${\upalpha }_{{\text{d}}}$(T) from Eq. (25) and plotted against frequency along with the original intensity. Also, the values of original and reconstructed intensities for the dipole spectrum are listed in Table 4.

**Table 4:** All the values of original and reconstructed intensities for dipole are listed.

Frequency (× 10^10^ Hz)	Original intensity (× 10^−21^ W Hz^−1^ m^−2^)	Reconstructed intensity (× 10^−21^ W Hz ^− 1^ m ^− 2^)
6.81	4.58	4.27
9.54	7.70	7.50
12.24	11.06	10.69
17.70	15.34	15.28
21.78	16.45	16.28
27.24	14.90	14.8
36.75	9.10	9.04
44.91	4.92	4.77
51.72	2.75	2.52
63.99	0.45	0.68

Using Eq. (16) the reduced chi-square ($\frac{{{\upchi }^{2} }}{NDF}$) is calculated to be 1.41.

The resulting temperature after mixing for dipole; ${\text{T}}_{{{\text{new}}}}^{{{\text{dipole}}}}$ = T [1 + $\left( {\frac{{{\Delta T}}}{{\text{T}}}} \right)^{2}$] = 2.748 K. There are several blackbodies at various temperatures. The blackbodies are mixed together, which distorts the original spectrum. Using the same formula as in monopole; the order of µ and y distortions are calculated as 2.7 × 10^−2^ and 1.3 × 10^−2^ respectively.

A graphical comparison is done between ${\upalpha }_{{\text{m}}}$(T) and ${\upalpha }_{{\text{d}}}$(T) and their resultant value ${\text{L(t)}} = \frac{1}{2}{\upalpha }_{{\text{m}}} \left( {\text{T}} \right) + \frac{1}{2}{\upalpha }_{{\text{d}}} \left( {\text{T}} \right)$.

## Discussion

The process of increasing the energy of radiation due to the encounter of electrons of hot galaxy clusters with CMB photons is called the inverse Compton effect. The Comptonization Parameter y explains how inverse Compton scattering affects the CMB. Due to the doppler shift of the CMB, the signals appear as a frequency-dependent distortion of the temperature dipole^37^. But here we have not considered the distortion due to the relative motion between the galaxy and CMB. We calculated the dipole temperature and distortion due to the mixing of blackbodies. This means the mixing of blackbodies has some contribution to dipole along with the doppler shift.

The value of deviation $\Delta {\text{T }} = \frac{{\text{V}}}{{\text{C}}}{\text{ T}} = { 3}.{3 } \times { 1}0^{{ - {3}}}$ K where T = 2.728 K and v = 370 km/s. The dipole is revealing the solar system appears to have a velocity of approximately 370 km/s relative to the local group of galaxies that implies that the local group of galaxies has a velocity of about 630 km/s relative to the rest frame of the universe^38^ This small deviation in temperature (3.3 × 10^−3^ K) is due to the doppler shift due to the velocity of the observer w.r.t the universe. But here we get the deviation as ± 0.270 K. Beyond T ± 0.018 K there is a contribution from the dipole.

**Figure 4:**
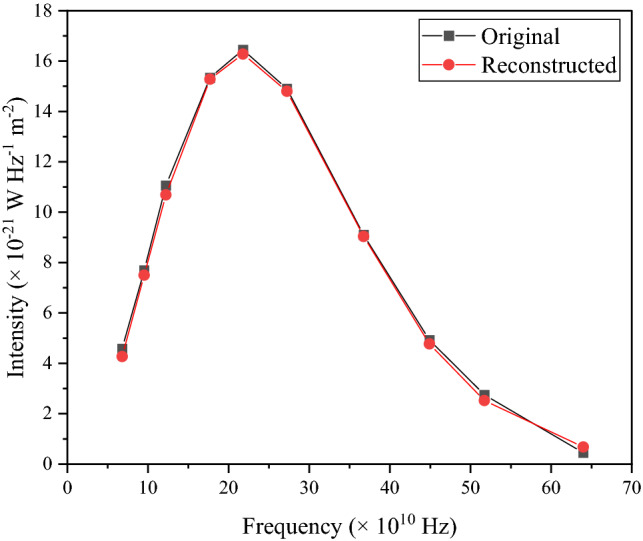
The original input intensity data for the dipole spectrum is reconstructed using the obtained probability distribution of temperature ${\upalpha }_{{\text{d}}}$(T). Plots of experimental and reconstructed intensities versus frequency are shown.

**Figure 5:**
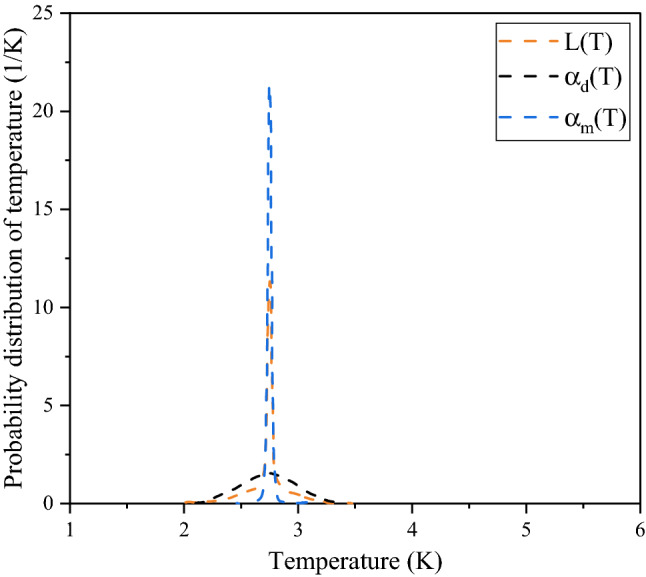
Probability distribution of temperature from a monopole, dipole and their results are plotted against temperature.

Figures 2 and 4 validate the method as the reconstructed intensities closely match the original intensity. It is evident that this method and choice of probability distribution function can faithfully reconstruct the original data. Figure 5 shows the comparison graph taken in our chosen range and the distribution is symmetric for monopole and dipole.

Here we have used the data set of COBE/FIRAS which has limited sensitivity and can measure the distortions up to 10^−5^ orders. Two upcoming projects PIXIE^39^ and PRISM^40^ aim to obtain the distortions more accurately with 10^3–^10^4^ times better sensitivity than COBE/FIRAS. The better sensitivity will be able to measure the small-scale fluctuation more significantly which will be able to give a clearer view of the origin of our expanding universe.

## Summary

In this paper, from both monopole and dipole spectrum, the probability distribution of temperature is obtained by employing blackbody inversion method. The temperature and its uncertainty are calculated by using the probability distribution of temperature. We also sought to reconstruct the monopole and dipole intensity by using the probability distribution of temperature. The concept of mixing weighted blackbodies is well interpreted. The spectral distortions like y and µ are calculated and found to be in the order of 10^−5^ and 10^−4^ respectively for the monopole spectrum and 10^−2^ for the dipole spectrum respectively. The method of BRI can also be extended for studies related to chemical potential and the fundamental properties of photons and radiation.

## Data Availability

The data sets used and/or analysed during the current study are available from the corresponding author upon reasonable request.
